# Measuring and Improving User Experience Through Artificial Intelligence-Aided Design

**DOI:** 10.3389/fpsyg.2020.595374

**Published:** 2020-11-19

**Authors:** Bin Yang, Long Wei, Zihan Pu

**Affiliations:** ^1^School of Design, Jiangnan University, Wuxi, China; ^2^Zhejiang Province Key Laboratory of Smart Management & Application of Modern Agricultural Resources, Huzhou University, Huzhou, China

**Keywords:** user experience, artificial intelligence aided design, human computer interaction, mobile application design, deep neural network, usability evaluation

## Abstract

This paper aims to propose a methodology for measuring user experience (UX) by using artificial intelligence-aided design (AIAD) technology in mobile application design. Unlike the traditional assistance design tools, AIAD focuses on the rational use of artificial intelligence (AI) technology to measure and improve UX since conventional data collection methods (such as user interview and user observation) for user behavior data are inefficient and time-consuming. We propose to obtain user behavior data from logs of mobile application. In order to protect the privacy of users, only a few dimensions of information is used in the process of browsing and operating mobile application. The goal of the proposed methodology is to make the deep neural network model simulate the user’s experience in the process of operating a mobile application as much as possible. We design and use projected pages of application to train neural networks for specific tasks. These projected pages consist of the click information of all users in the process of completing a certain task. Thus, features of user behavior can be aggregated and mapped in the connection layers and the hidden layers. Finally, the optimized design is executed on the social communication application to verify the efficiency of the proposed methodology.

## Introduction

With the development of mobile Internet, more and more users get information and services through mobile devices. Mobile devices represented by touch-screen mobile phones and tablet computers are gaining people’s favor with their high-density human–computer interaction (HCI). In these mobile devices, a variety of applications are being introduced into different categories, such as social transport letters, health care, and lifestyle, etc. Currently, there may be dozens or even hundreds of applications on mobile devices. However, mobile applications are becoming more and more homogeneous. How to achieve differentiated competition is a problem faced by many enterprises. At the same time, enterprises are trying to find a breakthrough. More and more enterprises begin to realize that user experience is closely related to user loyalty. Therefore, improving the user experience of products is the best business opportunity for enterprises (Park and Woo, 2006).

The concept of user experience has been widely spread and rapidly accepted in the field of HCI. It is generally believed that the concept of user experience was proposed and promoted by Norman (2002) in the early 1990s. Its connotation and framework have been expanding, involving more and more fields, such as psychology, HCI, and usability testing, which have been included in the relevant fields of user experience (Hassan Basri et al., 2016). User experience (UX) emphasizes the non-utility aspect of HCI and focuses on the user’s emotion, feeling, and the significance and value of such interaction in daily life. As a result, UX is seen as a desirable thing, although what it really means is still open and controversial (Law et al., 2009). Although more and more people accept and recognize the importance of UX, there is no consensus on the definition of user experience. Rajanen et al. (2017) discussed the views of UX professionals on definitions of usability and UX. They compared the research results of different countries and social–cultural groups. There are differences in the definition of user experience among user experience professionals, and there are systematic differences related to social and cultural conditions. UX professionals in Finland and France tend to emphasize the definition of experience qualities, while Turkey and Malaysia tend to reflect the definition of ease of use, utility, attractiveness, and usage. Experience is the user’s subjective psychological feeling, but the feeling will leave traces. Researchers can describe and measure them through objective evidence or experiments. The ISO 9241-210 defines UX as:

• “Person’s perceptions and responses that result from the use and/or anticipated use of a system, product or service.”

User experience focuses on the individual experience in relation to the use of a product.

Rajanen et al. (2017) believed that usability is now an established concept among UX professionals. Kocaballi et al. (2019) reviewed the understanding of UX in conversation interface literature and examines six questionnaires commonly used to evaluate conversation systems, in order to evaluate the potential applicability of these questionnaires in measuring different UX dimensions in this context. On the other hand, with a good usability of the product, users will feel convenient, fast, and comfortable after use and reduce the possibility of user operation error. Obviously, it will arouse users’ good emotional experience, make users feel happy, and then achieve the purpose of improving UX. In this work, we follow the definition of UX in ISO 9241-210, and strive to perceive and improve user experience in the process of using mobile applications. To better understand when to use which UX research methods, Christian Rohrer illustrated 20 popular methods in a three-dimensional framework (Rohrer, 2014). The UX research method proposed in this paper can be divided into behavioral and quantitative dimension in Rohrer (2014). Social platforms have gradually shifted to the mobile Internet, and several large and comprehensive social APPs (WeChat, QQ, and Weibo, etc.) have monopolized the market in China. Different APPs were designed to meet different social communication needs. Vertical social APP has the characteristics of strong pertinence and clear service field. People need not only one to two large social APPs, but also some vertical social APPs to meet single-point needs. Therefore, how to grasp the precise vertical users is an important issue. For example, Chinese users tend to focus on one or several applications to complete specific tasks based on mobile terminals. Users will have a strong sense of control over the overall status and distribution of task operations. This mode of application tends to combine multiple tasks into a single task process, thus freeing up some cognitive resources for users to use when they operate other tasks at the same time. If the subtasks in the “big and complete” application are less relevant, and the task process is not cohesive, users will compete for psychological resources, which leads to users wandering and switching between multiple tasks, and the overall efficiency is unpleased. It is a great challenge for a vertical social APP to develop rapidly and enhance its user loyalty under the environment of a large and comprehensive social APP monopoly market. “Waterman” is a vertical social APP belonging to water supply and drainage industry. However, the user experience and user loyalty of this APP is unpleased. We then optimized the application by the proposed methodology. The basic idea of the artificial intelligence-aided design (AIAD) methodology is to make the deep neural network model simulate the user’s experience in the process of using some functions. The contribution of our work can be summarized as follows:

• We propose to measure UX from the click behavior of users when they operate the application.
• According to the three levels of human brain activity, the corresponding machine experience model (MEM) is established.
• The neural network model is trained for specific tasks with projected pages. All features of user behavior can be aggregated and mapped in the connection layer and the hidden layer.

## Related Work

In order to make the best design of products in the early stage of product development life cycle, many observation methods have been introduced to capture and measure user experience. Battarbee and Koskinen (2005) effectively classify user experience methods into three categories: measurement, emphasis, and pragmatism. Measurement methods focus on all aspects of the user experience, which can be directly measured through physical reactions or subjective reports of the body. The emphasis method is to have a rich understanding of the user’s needs, wishes, dreams, and motives in the design stage through various formal methods, including visual and text data, as well as creative tasks. These methods aim to plan the future user experience and motivate designers, rather than evaluating the current user experience of the system. Pragmatic methods provide a holistic view of user experience, focusing on the understanding that the interaction among users, technology, and environment is an integral part of experience. Mctear et al. (2016) defined the interactive interface as the communication between users and machines through a variety of interaction technologies. They believe that with the rapid development of artificial intelligence (AI) technology and the rapid progress of semantic web, a large amount of online knowledge will emerge. Inspired by currently measuring and understanding methods based on AI techniques, we believe that user experience can be learned by machine. In this section, we briefly introduce the related works from three aspects.

### Measuring User Experience

Since the 1930s, traces of behavior have been collected in psychological research (Dumais et al., 2014). In this method, participants were asked to randomly stop, and their experiences were recorded in real time. AttrakDiff (Hassenzahl et al., 2003) is one of the most commonly used standardized questionnaires in HCI to measure hedonic quality. Although it explicitly focuses on hedonic quality, it also measures practical quality and the overall appeal of the product. According to the user experience model of Hassenzahl, (2005), AttrakDiff has a strong theoretical basis. The model considers that a product can have two main qualities: hedonism and practicality. Hedonic quality refers to the ability of product support to achieve the goal, while pragmatic quality refers to the ability of product support to achieve the goal. It consists of 28 items in three categories: pragmatic quality, hedonic quality, and attractiveness. It is worth noting that the theoretical model behind AttrakDiff does not attempt to measure emotions such as pleasure, satisfaction, happiness, or anger because they are considered to be the result of the cognitive assessment process Karapanos et al. (2009) developed a daily reconstruction method (DRM) to investigate the rich quality experience of users, and expound the concept of user experience in some narrative terms. They put forward an in-depth, 5-week ethnographic study that tracked six people during the actual purchase of the apple iPhone. They found that the motivation for long-term use was different qualities rather than providing positive initial experience.

However, traditional methods of user data collection and user feature model extraction are inefficient. With the increase in the amount of user data, the cost of research and development is also greatly increased. Meanwhile, AI technologies such as big data and machine learning had rapidly developed (Abualigah et al., 2018b; Abualigah, 2020b). Using these tools to assist in the design may be an efficient way. Dumais et al. (2014) summarized different types of behavior data for improving a design in Table 1. They believe that log files can be used to understand how users experience a product or service. Behavior log is the trace of human behavior seen through the sensor lens that captures and records the user’s activity. It ranges from low-level keystrokes to rich audio and video recording. User behavior data collection methods can be roughly divided as lab studies, field studies, and log studies.

**TABLE 1:** Different types of user data for improving design.

Types	Range	Experimental
Lab studies	Controlled interpretation of behavior with detailed instrumentation.	In-lab controlled tasks, comparison of systems
Field studies	In the wild, ability to probe for detail	Clinical trials and field tests
Log studies	In the wild, little explicit feedback but lots of implicit signals	A/B testing of alternative systems or algorithms

Logs also have the advantage of being easy to capture on a large scale. Although laboratory and field studies usually include tens or hundreds of people, journal studies can easily include data of tens or hundreds of millions of people. Such a large sample size means that even small differences between populations can be observed. In particular, large-scale logs provide an unusual but important piece of behavior data that is hard to capture in smaller studies. Log documents classify different types of user behavior data by some genetic algorithms. Genetic algorithms are usually used in information retrieval systems to enhance the information retrieval process Abualigah and Hanandeh (2015) applied genetic algorithms to text information retrieval. After that, a series of text clustering methods (Abualigah and Khader, 2017; Abualigah et al., 2017a,b, 2018a; Abualigah, 2018; Abualigah and Diabat, 2020a,b) were proposed by the author, which enriched and promoted the development of text clustering algorithms. In the next section, several classic click models based on user behavior logs data are introduced.

### Click Models

In recent years, more and more researchers are interested in how to use the data in the process of using software to better understand decision making. Click models have been wildly used to explain or predict the click actions of users (Jiang et al., 2020). Most of the click models are based on the most basic research on click models. It is believed that users browse search engines from top to bottom along the search results list. According to this assumption, the browsing order of users is consistent with the location order of search results. Most of the click models are based on location. In addition, the most important information source of click model is user interaction information (mainly click information), so the inference of user behavior and result correlation in the model is from click behavior. Therefore, these click models assume that all the results in the search page are homogeneous (all of them have similar forms, only different in content, corresponding to the model, only different in result relevance). After excluding the effect of result relevance, these results do not affect the user’s behavior.

Click logs can provide a valuable source of relevant information. However, the probability of click is affected by the position of the document in the result page, which brings deviation to the establishment of click model. Craswell et al. (2008) proposed a cascade model (CM) to handle such bias. They assume that users scan the search engine results page from top to bottom until they find a relevant document she clicked on. In its canonical form, CM assumes that “the clicked user will never return, and the skipped user will always continue,” which limits it to querying sessions with just one click. Different from CM models, Koller and Friedman (2009) developed the probabilistic graphical model (PGM). User behaviors on web search engines are represented as a series of observable and hidden events in PGM framework. It provides a mathematically reliable way to infer a group of events given some information about other events. Most probabilistic models distinguish two events: the user checks the document, and the user is attracted to the document. These events are generally assumed to be independent of each other. In addition, most models assume that a user will only click on a document if he wants to check it or is attracted to it. This problem has been addressed in the User Browsing Model (UBM; Dupret and Piwowarski, 2008). Based on the application of web search, user activity model can be divided into three categories: analysis model, whose purpose is to deeply understand the specific behavior of users and predict the future behavior model of users. UBM focuses the latter, which only uses the source information from web search logs. According to the ranking of documents and the distance from the last clicked documents (by ranking), UBM can estimate the inspection probability of documents. However, this method does not use maximum likelihood estimator and expectation-maximization algorithm to obtain the point estimation of the relevant parameters, but uses Bayesian method to infer their posterior distribution. Inspired by UBM, Liu et al. (2009) proposed a Bayesian browsing model (BBM), which has similar assumptions on user behavior. Two sets of experiments were presented to test model effectiveness and efficiency. The experimental results show that BBM has the ability of precise reasoning and is a single channel and parallelizable method.

Different click models have different models of test probability. The probability of attraction is calculated by different parameters (Koller and Friedman, 2009). However, the structure of the dependencies between events must be set manually. Different click models use different handmade dependency sets. Chen and Fischbacher (2016) discovered that simple information such as response time and click location can provide people’s preferred information. These data can be collected almost free of charge. They found that individualistic subjects click more often on their own payoffs than on the others’ payoffs. Moreover, the response time information and the click position information are complementary in explaining subjects’ preferences. Regular analysis of click locations is often used to optimize web design (Guo et al., 2009).

Previous work on click models has made a great effort in reducing the systematic bias and improving the trueness of relevance estimation by experimenting with different user behavior assumptions and building more sophisticated models. Click models aim to extract accurate relevance feedback from the noisy and biased user clicks. It is also important to test the reliability and accuracy of click model correlation estimation (Wang and Guo, 2017; Shen et al., 2018). A variety of information can be used to build a click model, such as information about the user, her current tasks, result presentation, result content, and other search characteristics. Shen et al. (2012) proposed a novel personalized click model to describe user-oriented click preferences. This model applies and extends matrix/tensor decomposition from the perspective of collaborative filtering, connecting users, queries, and documents together. This model is a general personalization framework, which can be incorporated into the click model. Although search click data is scarce, the model can penetrate query and document through potential eigenvectors, so as to deal with rare or even new query document pairs. Unfortunately, for many areas, even weak surveillance data can be scarce (Yang et al., 2018). The query generation system is trained on general domain data, but it is applied to target domain documents. This makes it possible to create any large, noisy, domain-targeted query document association pairs. Based on this, the zero-shot learning (Fu et al., 2015) technique can be used to synthesize the query-generated retrieval model (Huang et al., 2016). The click position cannot provide enough information for users to complete a task. There is a consistent relationship between response time and strength-of-preference, which arises from optimal solutions to sequential information sampling problems (Konovalov and Krajbich, 2016). Chen and Fischbacher (2016) investigated that the response time correlates with subjects’ preferences. The response times and click positions were used to infer people’s preferences. Therefore, response time is also used in our proposed framework.

Recently, Jiang et al. (2020) proposed a data-driven agent-based model to analyze the click position and user posting behavior in online review. The model explains how the click position affects the volume of posting main reviews and response reviews. It also analyzes the moderating effect of the number of items per webpage on the relationship between the clicking position and posting behaviors. They divided the clicking position into five modes. Because the posting behavior of the participant is driven by the knowledge and ability related to the reading content, the clicking position will affect the posting behavior of the members. Similar to the webpage on the monitor, clicking position can also be used in modeling user experience in mobile application design (Abualigah, 2020a).

Search engine page analysis through click model is another research direction in these years. A large number of search results including rich text information are introduced into search pages. These search results come from several sub engines with specific search targets, which are usually called vertical search engines. These vertical search results from the vertical search engine (such as the image results obtained by the image search engine) often have different presentation form from the traditional results, so the search results on the current search page are becoming very heterogeneous, which also makes the user’s browsing behavior habits and preferences may have great changes. By analyzing the large-scale search logs of a Chinese commercial search engine, Wang et al. (2013) found that more than 80% of the search result pages in the current Chinese search environment contain vertical results, and the vertical results in different forms of presentation have a great impact on the behavior of users, including the vertical results themselves (local impact) and the whole search page (global impact). Therefore, it is important to consider different vertical results. They conducted in-depth analysis on the change in users’ browsing behavior and finally summarized four user behavior bias assumptions: (1) attraction bias hypothesis, (2) global influence bias hypothesis, (3) first bias impact hypotheses, and (4) browsing order bias impact hypothesis.

### Measuring User Experience From Behavior Data

By assuming how the behavior bias affects the user’s click behavior, the click model can estimate the impact of behavior bias and the correlation between each operation separately. After training the click model of the log, the model can get the correlation estimation with small deviation and use it for the subsequent task prediction. For example, the click-based relevance estimation as ranking features can be used to train a learning-to-ranking model (Chapelle and Zhang, 2009). These features can also be used as weak supervision signals to train and test data-hungry neural ranking models (Li et al., 2019; Yang et al., 2020b).

One of the key problems in information retrieval is the location deviation. There are two problems with conventional methods. First, the information of the selected document is usually ignored when the user clicks on it. Second, they only consider the location deviation and ignore the other problems caused by users’ browsing behavior. In order to improve the performance of follow-up tasks, the correlation estimation given by click model should be as accurate as possible. Generally, the accuracy of estimation depends on two factors: authenticity and accuracy. Authenticity is the estimation of system error (i.e., estimation deviation), while accuracy is the estimation of random error (i.e., estimation variance). Mao et al. (2019) suggested to study the reliability of correlation estimates derived from click models. The posteriori distribution of correlation parameters is inferred by the method of variable decibels instead of the point estimation of correlation. Based on the posterior distribution, the reliability measure of point pair correlation estimation is defined.

In recent years, deep learning techniques have been successfully applied to image understanding and information mining tasks (Guo et al., 2015; Liu et al., 2015b; Zou et al., 2018; Cheng et al., 2019; Yang et al., 2020a). By using deep learning framework, deep neural networks (DNN) can mine high-level abstract information and even predict user behavior, such as human action recognition (Guo and Chen, 2015), VISL design (Guo et al., 2014; Huang et al., 2015; Liu et al., 2015a), classification (Luo et al., 2017), and saliency detection (Niu et al., 2018). Nair and Hinton (2010) used the pre-training method to alleviate the problem of local optimal solution and pushed the hidden layer to seven layers, which made the neural network have “depth” in the real sense and thus opened the upsurge of deep learning. In order to overcome the disappearance of gradient, transfer functions such as Relu and Maxout replace sigmoid and form the basic form of DNN (Yang and Ma, 2016). Subsequently, more and more user behavior analysis methods are proposed based on various of neural network models, such as recurrent neural network (RNN; Zhang et al., 2017), convolutional neural networks (CNN; Zhang et al., 2018; Zhu et al., 2018), long short-term memory (LSTM) networks (Borisov et al., 2016), etc.

Based on the idea of distributed representation, Borisov et al. (2016) proposed a neural click model for web search. Vector state is used to represent the information needs and available information of users. Vector state components are used to model user behavior. User behavior is modeled as a sequence of vector states associated with a query session. The query initializes the vector state, which is then updated iteratively based on the information that interacts with the search engine. One of the key problems of location deviation is to deal with implicit but biased user feedback data in information retrieval (Guo et al., 2018). Unbiased sorting usually relies on causal model, while debias obtains user feedback by reverse tendency weighting. Although these methods are practical, there are still two problems. First, when inferring a user’s click, the impact of contextual information, such as checked documents, is often ignored. Second, only the location deviation is considered, and other problems caused by users’ browsing behavior are ignored. Recently, Jin et al. (2020) adopted RNN to model the contextual information and estimates the conditional likelihood of user feedback at each position. Then, they combine the survival analysis with the probability chain to restore the correct joint probability of user behavior.

## Methodology

**FIGURE 1:**
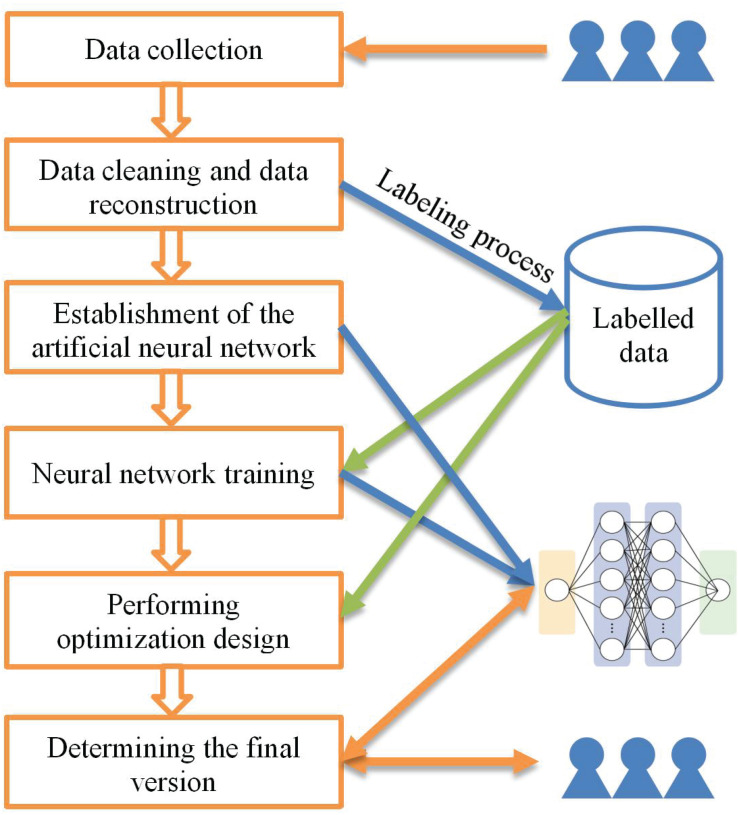
Flowchart of the proposed AIAD framework.

With the rapid development of AI technology, mobile application designs have also ushered in changes and development. As presented in Figure 1, several AIAD techniques were used in the optimization scheme. The proposed AIAD scheme is based on the following five steps:

1. First, a data collection function is developed and set into the former version of the software.
2. Second, data cleaning and data reconstruction processes are performed on the collected dataset. Successful operations are marked as positive records, and failed operations are marked as negative records.
3. Third, an artificial neural network is designed to adapt the specific work. The network structure (such as VGG, and DBN, etc.) can be selected and adjusted according to the task.
4. Fourth, the reconstructed data is input into the neural network model as training data. In this work, the clicks of all users in the process of completing a task are project into the pages needed for the task.
5. The user’s operation information mapping heat-map and the original APP interface.
6. Fifth, the optimization design is performed according to the labeled user’s data. For example, the designer can find some problems in function logic and interface design by browsing the user operation process of failure records.
7. Finally, the interface according with normal operation data of the optimized APP can be input into the trained neural network model. The neural network will give an evaluation of the optimization effect (e.g., give a score). In this way, the proposed AIAD system is more like a virtual “designer assistant” existing in the data.

### User Behavior Data Collection

**FIGURE 2:**
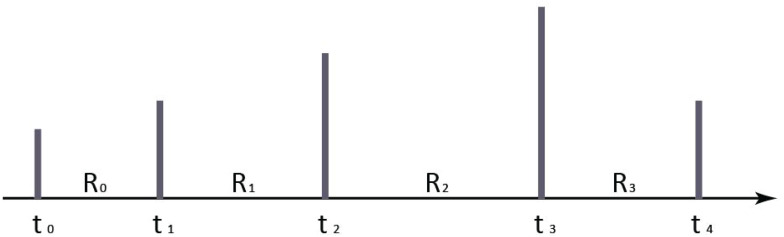
Simplified mental model.

Data collection is the process of gathering and measuring information on targeted variables in an established application. What types of user data should be collected from mobile applications? Most users may be reluctant to expose too much personal data. Thus, the fewer user data the program collects, the better. In our proposed data collection function, only two kinds of user behavior information were collected based on the theory of spatial consistency of mental model. Spatiality is an important feature of mental model for HCI. Spatiality plays an important role in the correct use of menu interface, search for information in hierarchical file management and navigation interface. Consistency in mental model space is important while designing a mobile application. During the whole process of a task, the logical consistency in mental space is caused by the material representation (icon, text, graphics, and layout mode, etc.) and feedback on different interfaces. This consistency includes size consistency, layout consistency, and texture consistency, etc. Figure 2 is a simplified mental model with spatial consistency of layout. The spatial consistency of mental model provides a good criterion for evaluating the rationality and performance of mental models. In this model, only several data (page, button, position of the click/drag, and operation time, etc.) are needed.

We can obtain detailed information of user’s behavior by projecting the coordinates of each click on the page. Therefore, only page ID, coordinates and retention time in page are collected. Table 2 presents some user behavior records for a mobile application of “Waterman.”

**TABLE 2:** User behavior records for searching friends on “Waterman.”

Task	Page ID	Coordinate 1	Coordinate 2	Retention time (ms)
Search friends	CD101	605, 1,775	605, 1,775	2,424
Search friends	CD102	988, 143	988, 143	412
Search friends	CD102	1,012, 148	1,012, 148	788
Search friends	CD103	1,175, 1,071	1,175, 1,071	231
Search friends	CD103	1,195, 1,063	1,195, 1,063	147
Search friends	CD103	1,199, 1,074	1,199, 1,074	88
Search friends	CD103	845, 1,442	878, 941	211
Search friends	CD103	812, 1,522	846, 874	189
Search friends	CD103	744, 578	744, 578	411

### Availability Evaluation for a Task

**FIGURE 3:**
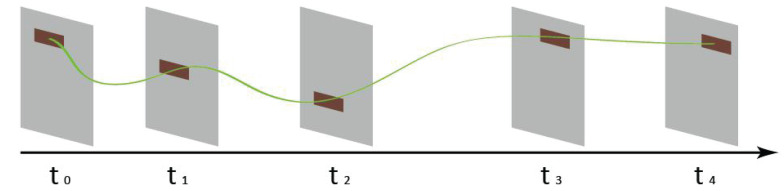
A histogram example of Figure 2.

A task can be divided into several units according to the correlation between its internal components in interactions. To evaluate the availability for a task performed by a user, an availability algorithm of interface interactive is proposed in this paper. We first mark each click event on the timeline for a task. Let *R*_i_ denotes the retention time of a page in a specific task. We weight *R*_i_ as the value of each click. We then obtain a histogram of timeline for a task. Figure 3 is a histogram example of Figure 2.

After superimposing and normalizing all histograms of the same tasks, we get an overlapping histogram. However, histograms cannot be directly superimposed because different operations have different timelines for a task. All histograms should be scaled into the same length. Therefore, we map all timelines into the same length by scaling projection algorithm. A scaled and superimposed histogram is then obtained and saved as an array data. All arrays are divided into positive and negative categories.

In order to project the clicks of all users in the process of completing a task *T*_i_ into the pages, the histogram data needs to be converted into image data. We use a circle with gray value α to represent each click. The gray value α can be roughly calculated by dividing 255 by the dwell time of each page. In practical application, the calculation method of gray value α can be adjusted to adapt to different neural network models.

### Optimization by Using Artificial Neural Network

**FIGURE 4:**
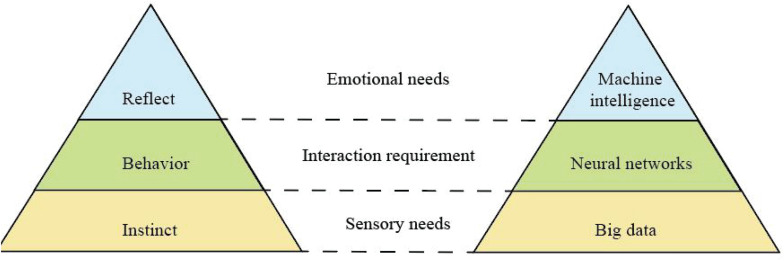
Human behavior model and proposed machine experience model.

Recurrent artificial neural networks are very helpful to solve dynamical problems. Compared to Shallow Learning, the architecture of artificial neural network contains more hidden layers. Norman (2002) proposed that human brain activity can be divided into three levels: instinct, behavior, and reflection. Based on these three levels and AIAD technology, we propose to establish a corresponding MEM, as shown in Figure 4.

**FIGURE 5:**
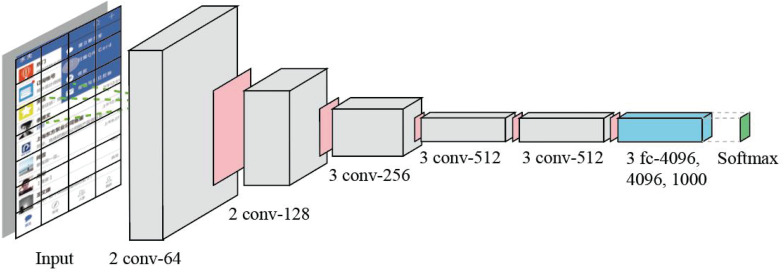
The deep neural network with 16 layers used in our methodology. The parallelogram with pink color is Max-pooling layer.

Big data mainly involves data distributed processing, storage, mining, and other technologies, which represent the ability of large-scale data processing and is a good support for deep learning. Deep learning supported by massive and valuable data has become more accurate and intelligent. By analyzing the multilevel mapping and association mechanism of data, neural network, and machine intelligence, the logical relationship among intuition layer, behavior layer, and reflection layer can be mapped. In this work, we use a classic neural network architecture VGG (Simonyan and Zisserman, 2014) as our deep learning model. The reason why we choose VGG is that its structure is very simple, and it is easy to be implemented by non-professionals. The size of convolution kernel (3 × 3) and the maximum pooling (2 × 2) layer used in the whole network are the same size. The combination of several small convolution layers is better than that of a large convolution layer. User behavior data from mobile operation is mapped on the corresponding page. A classic VGG with 16 layers is presented in Figure 5.

Long short-term memory is used to construct the network model, since the task has great time attribute. LSTM is a kind of special RNN, which is mainly used to solve the problems of gradient vanishing and gradient explosion in the process of long sequence training. RNN is a kind of neural network for processing sequence data. Compared with the general neural network, it can process the data, which change according to the time series. Inspired by the LSTM-CNN Model (Tan et al., 2015), we propose to combine the LSTM model with the VGG network structure. The training process of artificial neural network can be summarized as the following steps.

First, we divide the training data into task groups according to different task *T*_i_ (for example, search friends) of an APP. In this way, each group of tasks contains the pages that must appear, which are sorted in the order in which they appear.

**FIGURE 6:**
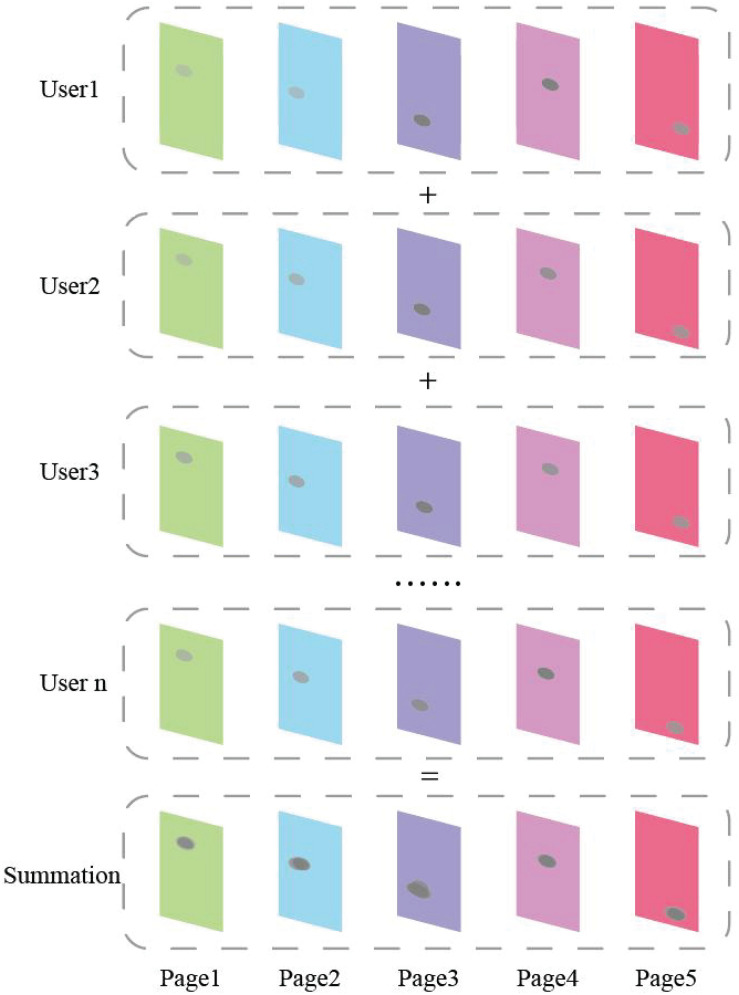
An example of the proposed projection method.

Second, we project clicks of all users in the process of completing a task *T*_i_ into the page needed for the task, as described in the Availability Evaluation for a Task section. Figure 6 is an example of the proposed projection method. The projected page groups of successful tasks are marked as positive samples, while failed tasks are marked as negative samples.

Third, we train the neural network model according to the specific task. Each projected page in the task group is used as network input. Thus, all the features will be aggregated and mapped in the connection layer and the hidden layer.

The deep neural network has only one output representing the score of input data. Since the neural network model has been trained on the user behavior data from the previous version of APP, the model can more reflect the user’s cognition and behavior habits, and then, the designer finds out the what need to be improved based on the user’s click behavior data, and improves the interface and function path accordingly to obtain an optimized version. The interface according with normal operation data of the optimized APP can be input into the trained neural network model. The neural network will give an evaluation of the optimization effect. In this way, the proposed AIAD system is more like a virtual “designer assistant” existing in the data. The speed and efficiency of APP development and improvement will be greatly improved.

## Discussion

User experience can be defined as an overall experience, which includes all aspects of user interaction with products or services. All existing theories about user-centered design, availability, impact engineering, and technology acceptance model are applicable to UX (Park et al., 2013). Traditional interaction design process, design principles, design patterns, and other methodologies are applicable to the design of traditional HCI products or services. For the application of intelligence and situational awareness, they are generally applied in a wide range without pertinence.

In order to verify the effectiveness of the proposed methodology, we evaluate a classic experiment to compare the usability of the optimized mobile application. It is suggested that 80% of usability problems could be exposed by five to seven participants in usability testing. Therefore, we invited six users to participate in the evaluation with their own mobile devices. These users are school students who have mobile application experience and use mobile social application services, including three boys and three girls, distributed in different grades and majors. Before the experiment, we explained to each participant the purpose of the experiment and the type of data that will be sent to the mobile phone. The whole experimental study lasted for about 2 months. Users were required to install a “Waterman” application on their mobile phones. User’s log data and user evaluation documents were obtained and stored. Because the screen of mobile terminal is small, it can only present content in limited space, so it is important to present users a sense of visual comfort. So, the esthetics of interface should also be included in the evaluation index. Finally, we determined seven indicators to evaluate the usability of “Waterman,” namely, learning, effectiveness, efficiency, error, interface esthetics, and satisfaction. As shown in Table 3, we designed four experimental tasks as examples.

**TABLE 3:** Four tasks designed in the experiment.

Tasks	Operation process
User registration	(1a) Open sub-menu “Mine.” (1b) Run application for the first time. (2) Choose “Login or Register.” (3) Input telephone NO. (4) Input user information (e.g., username, gender, company, sub-segments). (5) Submit.
Add a new friend	(1) Open sub-menu “Connections.” (2a) Choose “New friend.” (2b) Click button “+.” (3a) Input a friend’s account. (3b) Find friends nearby. (4) Browse friends’ information (e.g., username, gender, company, sub-segments). (5) Submit add friend request.
Search friends	(1) Open sub-menu “Connections.” (2a) Browse the list of friends and select one. (2b) Click “Find” button and input a friend’s name. (3) Open the page of the friend you want to find. (4) Call him.
Participate in activities	(1) Open sub-menu “Finds.” (2) Browse the list of activities. (2a) Filter some activities. (3) Select an activity and open the detail page. (4) Choose to attend this activity. (5a) Set an alarm.

In the evaluation process, each participant needed to complete all tasks and submit the feedback feeling every 3 days at least. All the user behavior data in the process of application operation were uploaded to the server and stored as log files. The evaluation results are presented in Table 4.

**TABLE 4:** Evaluation results.

Evaluation indicators	The former version	The optimized version
Learning	2.4	3.0
Effectiveness	3.4	3.5
Efficiency	1.8	3.8
Error	2.3	4.3
Interface esthetics	3.1	3.5
Satisfaction	2.2	4.3

*Note. Indicator values range from 1 to 5 (5 for best).*

As shown in Table 4, the optimized mobile application was superior to the former in all indicators. Evaluation results verified the effectiveness of our proposed methodology, especially in efficiency, error, and satisfaction. We believe that deep neural network can understand user experience better through behavioral data. With the assist of deep network model trained by user behavior data, the version can be determined efficiently and accurately in A/B test. Specifically, two versions of APP (version A and version B) were used in the experiments of two groups of subjects. The results of the evaluation are then recorded and used to determine which version is better. Experimental results show that the efficiency of optimal design has been greatly improved. The main difference between this algorithm and other algorithms is that we establish the relationship between human brain activity and machine experience model. We propose to measure UX from the click behavior of users by deep neural network model. Thus, all features of user behavior can be aggregated and mapped in hidden layers. The evaluation results verify the effectiveness of the AIAD scheme. However, for some indicators (such as learning, effectiveness), deep neural network is unable to greatly improve the user experience. This means that AI is not omnipotent; it can only be used as an assistant design tool by designers.

## Conclusion

Designers are lagging behind in taking advantage of this not so new technology. The future of AI mediation seems to be driven by data availability and learners’ performance rather than a well-thought out user-centered vision. At present, AI technology has rarely become a standard part of user experience design practice, nor a part of design pattern, prototype tool, or design education. Yang et al. (2016) think that user experience designers still lack the knowledge of deep neural network. In this paper, we present a methodology for understanding and measuring user experience by using AI techniques in mobile interaction. The key of AI technology is using machine learning, more specifically, using deep neural network. The assumption of this paper is that by analyzing the multilevel mapping and association mechanism of data, neural network, and machine intelligence, the logical relationship among intuition layer, behavior layer, and reflection layer can be mapped. Deep neural network model can simulate user experience to a certain extent based on user behavior data. Therefore, we propose to make full use of user’s log data to reduce the acquisition cost of user behavior data. The flexibility of this model makes it good assistant design tool, which can be used in future work to understand the user’s experience.

For future work, first of all, we hope to extend our method to more user behavior data, such as drag and return. Second, we want to incorporate information about the user, e.g., user profile, location, and time, etc. Third, the deep neural network model for matching user mental model is not limited to VGG; we will try to adjust or improve the network structure.

## Data Availability Statement

The raw data supporting the conclusions of this article will be made available by the authors, without undue reservation.

## Ethics Statement

Ethical review and approval was not required for the study on human participants in accordance with the local legislation and institutional requirements. The patients/participants provided their written informed consent to participate in this study.

## Author Contributions

BY was in charge of the study design. LW was responsible for the literature review. ZP was in charge of the coding and assessment. All authors contributed to the article and approved the submitted version.

## Conflict of Interest

The authors declare that the research was conducted in the absence of any commercial or financial relationships that could be construed as a potential conflict of interest.
